# Health Professionals’ Preferences for Next-Generation Sequencing in the Diagnosis of Suspected Genetic Disorders in the Paediatric Population

**DOI:** 10.3390/jpm15010025

**Published:** 2025-01-10

**Authors:** Mario Cesare Nurchis, Gerardo Altamura, Gian Marco Raspolini, Enrico Capobianco, Luca Salmasi, Gianfranco Damiani

**Affiliations:** 1 Department of Life Science, Health and Health Professions, Università degli Studi Link, 00165 Rome, Italy; 2 Section of Hygiene, Department of Health Science and Public Health, Università Cattolica del Sacro Cuore, 00168 Rome, Italy; gerardoandrea.altamura01@icatt.it (G.A.); gianmarco.raspolini01@icatt.it (G.M.R.); gianfranco.damiani@unicatt.it (G.D.); 3 The Jackson Laboratory, Department of Computational Science, Farmington, CT 06032, USA; enrico.capobianco@gmail.com; 4 Department of Economics and Finance, Università Cattolica del Sacro Cuore, 00168 Rome, Italy; luca.salmasi@unicatt.it; 5 Department of Woman and Child Health and Public Health, Fondazione Policlinico Universitario A. Gemelli IRCCS, 00168 Rome, Italy

**Keywords:** paediatric population, NGS tests, suspected genetic disorders, discrete choice experiment, health policy

## Abstract

**Background/Objectives:** Next-generation sequencing (NGS) can explain how genetics influence morbidity and mortality in children. However, it is unclear whether health providers will perceive and use such treatments. We conducted a discrete choice experiment (DCE) to understand Italian health professionals’ preferences for NGS to improve the diagnosis of paediatric genetic diseases. **Methods:** The DCE was administered online to 125 health professionals in Italy. We documented attributes influencing professionals’ decisions of NGS, including higher diagnostic yield, shorter counselling periods, cost, turnaround time, and the identification of fewer variants of unknown significance. **Results:** Results show that factors such as higher diagnostic yield, shorter counselling periods, lower costs, and faster turnaround times positively influenced the adoption of NGS tests. Willingness to pay (WTP) estimates varied from EUR 387 (95% CI, 271.8–502.9) for 7% increase in the diagnostic yield to EUR 469 (95% CI, 287.2–744.9) for a decrease of one week in the turnaround time. Responders would reduce diagnostic yield by 7% to decrease the turnaround time by one week in both the preference and the willingness to trade (WTT) spaces. Respondents prioritised diagnostic yield (RI = 50.36%; 95% CI 40.2–67.2%) compared to other attributes. **Conclusions:** therefore, health professionals value NGS for allowing earlier, more accurate genetic diagnoses.

## 1. Introduction

Clinical genetics is an evolving speciality influenced by the availability of increasingly sophisticated investigational techniques [1]. Due to technological development, genetic counselling is gaining importance with further applications of genetic testing for diagnosis and clinical treatment [2]. More than 77,000 genetic tests [3] were estimated to be in use in 2022, with many others being developed and focused on detecting mutations in genes, chromosomes, or proteins. Over the past 50 years, DNA-based tests have shown an exponential increase in the number of disorders for which genetic testing has become available (biochemical, chromosomal, etc.) [4]. DNA and RNA sequencing and variant/mutation detection [5] via next-generation sequencing (NGS) have been integrated into molecular pathology and significantly increased the breadth of genomic information through the profiling of hundreds of genes [6,7], especially in the oncological field [8].

It is possible to distinguish three typologies of NGS testing: whole exome sequencing (WES), whole genome sequencing (WGS), and targeted NGS sequencing or panel NGS sequencing [9]. Targeted NGS is the most employed sequencing approach for molecular analysis in clinical practice [10]. Conversely, the widely used WES and WGS have limited clinical applications due to their low coverage depth and relatively high cost [11,12]. Studies demonstrate that these procedures can shorten the differential diagnosis process, targeted treatment timing, and genetic and prognostic counselling [10]. Genetic diseases (as per single-gene disorders, genomic structural defects, and copy number variants) are a leading cause of death in children under ten years old [11,12,13,14,15]. The application of NGS testing to neonatal care for early diagnosis of congenital diseases is a promising field of application. The goal is to reduce the disease burden for neonatal conditions with significant clinical impact by identifying all genetic disorders to allow for the timely implementation of appropriate therapies, apart from symptoms [16].

Neonatal intensive care units (NICU) are a pivotal service for critically ill neonates experiencing high morbidity and mortality rates, with significant diagnostic errors accounting for up to 20% of autopsied deaths. Newborns with undiagnosed or rare congenital disorders may mimic severely ill ones with more commonly acquired conditions, meaning that the diagnostic evaluation context can generate unique biases leading to diagnostic errors [17]. However, NGS testing might partially solve misdiagnosed clinical status hindrances. Assessing the preferences of the professionals involved could play an essential role in determining which method should be introduced in clinical practice. Indeed, specialised healthcare teams in many countries had stringent control over patient access to such NGS tests [18]. The preferences and insights of these professionals may play a multifaceted role in shaping the future of genomics in healthcare. Health professionals possess a deep understanding of the clinical context, ensuring that any new genomic method aligns with the practical needs of patient care. Furthermore, their input is invaluable for strategies successfully implementing new methods and helping identify barriers and facilitators to adopting the NGS techniques. Ethical considerations, resource allocation, and public trust in genomics heavily rely on their expertise. By fostering collaboration, ensuring regulatory compliance, and promoting continuous improvement, the engagement of health professionals is essential for the responsible integration of genomics into public health practice.

Surveys are a high-quality, efficient, and viable tool for exploring healthcare professionals’ preferences and opinions about a specific topic [19]. To elicit preferences that can be used in the absence of revealed preference data, the most valid survey design is a discrete choice experiment (DCE). This method detects the stakeholders’ propensities towards different attributes of hypothetical alternative scenarios. At the same time, the responses are employed to determine whether the preferences are significantly influenced by the attributes and their associated relevance [20].

The DCE methodology and especially the willingness to pay (WTP) elicitation are well-established approaches within the clinical field. In the realm of genetic testing, Buchanan et al. determined the attributes encouraging or discouraging the uptake of genomic tests in the context of inherited cardiovascular disease in the UK. Findings show that healthcare professionals predominantly emphasise the importance of diagnostic yield and the capacity to discover fewer variants of unknown clinical relevance in contrast to the duration of counselling, which received comparatively less emphasis [21]. Ries et al. documented results concerning the WTP for genetic testing. In this study, individuals from the general population demonstrated a readiness to allocate more than CAD 500 towards genetic diagnostic services. Meanwhile, Dhanda et al. indicated that payer-preferred genetic tests, with a focus on enhancing quality of life, exhibited substantial consensus among health professionals for effecting modifications in medical care and extending life expectancy through precisely tailored treatments [22]. Albeit in a different research context, Leigh et al. elicited preferences and WTP estimates from health professionals about the adoption of mobile health services in the UK [23]. Similarly, Tarekegn et al. measured preferences of health professionals for human papilloma virus vaccines through the WTP approach, helping policy makers setting priorities among alternative cervical cancer prevention methods in poor countries [24].

This paper tried to fill a gap in favour of understanding attributes related to NGS techniques implementation in a health system (i.e., Italy) where there is very little prior evidence. This paper elicits individual preferences, the WTP, and the willingness to trade (WTT) for NGS techniques. Being that health professionals are the key decision makers in the diagnostic workflow, stated preferences and the analysis of their WTTs contribute to steering NGS techniques adoption within the clinical practice by prioritising specific steps within the whole diagnostic process. Furthermore, from a policy perspective, given that our WTP estimates were elicited in the absence of NGS techniques regulations, they may represent shadow prices and thus are of paramount relevance for policymaking in defining tailored reimbursement tariffs that may be subsequently adopted for ad hoc economic analyses by decision makers.

This study aims to assess health professionals’ preferences regarding the attributes of NGS techniques for the diagnosis of suspected genetic disorders by using a DCE approach.

## 2. Materials and Methods

DCEs elicit individuals’ stated preference parameters between two alternative treatments. According to the Lancasterian theory of demand, treatments are defined by their characterising attributes [25], while the alternatives are generated by changing the values taken by a set of attributes. Generally, each individual is asked to choose the option from a list of choice sets, thus obtaining multiple observations [26]. Moreover, the inclusion of continuous variables, such as cost or waiting time, allows researchers to compute the willingness to pay (WTP) [27,28] or willingness to trade (WTT) [29,30,31,32] for variations in attribute levels.

Considering the abovementioned measures as important preference parameters useful in interpreting DCE results, our focus was on identifying a set of attributes and their importance levels according to health professionals. Then, both the relative importance of the attributes and the WTP/WTT were estimated by asking health professionals to choose between two NGS techniques with different test attributes in multiple-choice tasks. As a note, institutional review board approval for this study was obtained from the Ethics Committee of the Fondazione Policlinico Universitario Agostino Gemelli IRCCS (protocol code 4952; date of approval: 19 May 2022). The study adopted anonymous data only, which guaranteed full adherence to the Helsinki Declaration of Ethical Principles and with Italian (Law 2003/196) and international (EC/2016/679) data protection regulations. Written informed consent was obtained from all participants prior to their inclusion in the study. Participants were informed about the purpose of the research, the voluntary nature of their participation, and the measures in place to ensure the confidentiality and anonymity of their responses.

### 2.1. Sample Population

The sampling population selected for this experiment was composed of health professionals working in highly specialised centres in Italy and with expertise in using NGS techniques to diagnose the paediatric population with suspected genetic disorders. The DCE survey was circulated among the health professionals of twelve Italian hospitals with expertise in medical management and/or genetic counselling of genetic diseases to guarantee that the sampling population reflected standard characteristics (e.g., age, gender) and preferences.

The sample size was calculated according to the formula proposed by Johnson and Orme [33,34]:

$$N=\frac{500\times c}{\left(t\times a\right)}.$$

This rule of thumb suggests that the sample size depends on the number of choice tasks (t), the number of alternatives (a), and the largest number of levels for any of the attributes (c). A more analytic approach by de Bekker-Grob et al. [35] was adopted to look for the minimum sample size for testing the significance of the various parameters with a desired power level. The following formula was applied:

$$N_{k}>{\left[\frac{\left(Z_{1-a}+Z_{1-b}\right)se\left({\bar{\beta }}_{k}\right)}{{\bar{\beta }}_{k}}\right]}^{2}$$

where $se\left({\bar{\beta }}_{k}\right)$ is the asymptotic standard error of the parameter $\hat{\beta_{k}},$ *a* is the significance level (i.e., 95%), and *b* is the power level (i.e., 80%), while $Z_{1-a}$ and $Z_{1-b}$ represent the 100 (1 − *a*)th and 100 (1 − *b*)th quantiles of the standard normal distribution, respectively.

### 2.2. Selection of Attributes and Levels for Each Testing Alternative

Attributes and levels in Table 1 were defined by adopting the approaches suggested by Regier et al. [36].

Literature was extensively reviewed by querying the primary scientific databases (i.e., PubMed, Scopus, EMBASE, and Web of Science) to identify the studies providing information on NGS-related factors that health professionals consider relevant to the diagnosis of suspected genetic disorders (see Supplementary Text S1 for additional details). Then, 12 potential attributes were chosen and brought to interviews with health professionals such as paediatricians, medical geneticists, biologists, and laboratory scientists, who rated the importance of each attribute (i.e., diagnostic yield, turnaround time, counselling time, ability of the test to identify variants of unknown significance, test cost, ease of interpretation for clinicians, follow-up testing requirements, ease of interpretation, security measures, sample requirement, reanalysis option, and clinical actionability). Five attributes were selected based on the rank assigned, the clinical and scientific plausibility, and the ability to capture the key characteristics of the testing practice. Finally, levels were defined for each attribute following the results of the interviews and the literature review.

### 2.3. Experimental Design

A full factorial design adopting the attributes and levels hitherto defined resulted in 675 potential choice sets (given by the multiplication of the attributes with five and three levels, 5^2^ × 3^3^), which are excessive for a DCE being too time-consuming to be completed. According to McFadden and Train [37], completeness, monotonicity, and transitivity of preferences are essential conditions for interpreting the DCE parameters’ estimates. When full factorial design is not feasible, D-optimality is the standard metric for design construction [38]. Therefore, the design was restricted to 10 choice tasks adopting a D-efficient algorithm appointed to identify a list of choice sets in which dominant alternatives are absent, and choice sets are not repeated. The D-optimal designs approach was adopted to maximise the determinant of the information matrix. These designs consider factors such as main effects, interactions, and curvature to achieve the highest efficiency level. Only the main-effects design was used to avoid the complexity of the design, and the estimation of interaction effects among attributes was denied at the design stage. Python programming language was used to generate the D-optimal designs. Each participant was faced with 10 choice tasks.

### 2.4. DCE Survey Design

In each choice set, respondents were presented with the same hypothetical scenario. This scenario described a situation in which a newborn came to the specialist’s attention with muscular hypotonia and a negative muscular spinal atrophy test. Once brought to the neonatal intensive care unit for not feeding and irregular breathing, routine controls confirmed low control of the head and hypotonic arms, leading the health professional to a potential genetic origin of the clinical condition. Respondents were asked to choose the two alternatives (genetic test A or genetic test B) they would prefer in each set. The DCE was set up in an unlabelled and forced choice format. Supplementary Text S2, in the Supplementary Materials, shows a hypothetical choice set presented to respondents and a description of each attribute. Similar evidence already available in the scientific literature [37,38,39] showed that an opt-out alternative was excluded since individuals were asked which option they would prefer rather than which one they would have chosen. Moreover, given the clinical complexity of the investigated population, healthcare professionals cannot avoid the conduct of an NGS test. Particularly, the decision to exclude an opt-out option in this DCE is grounded in the realistic simulation of clinical decision-making scenarios at tertiary care facilities involved in the survey. In this context, children presenting with a suspected genetic disorder are offered one of the two advanced genomic tests. The choice does not typically include traditional genetic testing or the option to forego testing altogether. The final DCE survey was organised as follows: (a) the welcome page provided respondents with instructions on how to complete the survey, the legal statements about personal data confidentiality, and the declaration of informed consent; (b) the respondents completed the 10 choice tasks; and (c) the DCE survey ended with multiple choice questions regarding the respondents’ demographic and background information.

### 2.5. Data Collection

A pilot survey was conducted among the study team and local collaborators. A few presentational changes were made, and some survey text was edited for clarity. No concerns were raised about the experimental design. Data were collected through an online survey using Qualtrics (Qualtrics, Provo, UT, USA) from 1 June 2022 to 31 August 2022 (92 days). Respondents were contacted electronically via email to invite them to complete the DCE.

Missing data and straight-lining responses were systematically addressed to ensure the reliability of the study findings. Missing data, defined as unanswered choice sets, were addressed through listwise deletion. Straight-lining responses, identified as instances where a respondent consistently selected the same alternative across all choice sets, were excluded from the analysis as these likely reflect disengagement or lack of understanding of the task.

### 2.6. Econometric Analysis

#### 2.6.1. Utility Function

A random utility function approach was used to predict choices and compute preference weights from the data collected through the DCE survey design. According to this framework [39], the utility that a respondent assigns to each alternative can be written as $U_{ntk}=V_{ntk}+\varepsilon_{ntk}$, where $V_{ntk}$ is the deterministic part of the utility obtained by the respondent *n* choosing the alternative *t* in the choice set *k*. Given two scenarios (i.e., genetic tests A and B), the assumption is that a respondent will choose alternative A if $U_{nAk}>U_{nBk}$. Under this framework, we can assume that respondents make trade-offs among attribute levels to maximise their utility. The utility function can be written as follows:

$$U_{ntk}=\sum_{k=1}^{5}\beta_{nk}A_{ntk}+\varepsilon_{ntk}$$

where $\beta_{nk}$ are the preference weights of each attribute level, $A_{ntk}$ are the attributes, and $\varepsilon_{ntk}$ is the error term.

#### 2.6.2. Model

A mixed logit regression analysis was implemented to estimate the model of choice behaviour [40]. This approach allows consideration of preference heterogeneity among respondents by permitting one or more model parameters to be randomly distributed [41,42]. Furthermore, the mixed logit model allows for within-person correlation across choice tasks. When variables are random, it is necessary to specify a distribution function. In the present analysis, a log-normal distribution was attached to random parameters. Variables are considered fixed when they are not assumed to be random. We first estimate a model with all variables included as random, then, by looking at those with a non-significant standard deviation, we identify fixed variables and use this information to specify our final model. We assumed a linear relationship between attributes and choices and considered all attributes as continuous (the presence of non-linearity was tested by estimating a model with attributes expressed as dichotomous variables. We did not find evidence of a non-linear relationship between attributes and the outcome, and for this reason, we decided to consider attributes as continuous variables) variables. Including a variable measuring costs allowed the estimation of the WTP both in preference and WTP space [42]. WTP assigns monetary values to attributes (i.e., how much money respondents are willing to pay for a one-unit improvement in one of the attribute levels [43]). Estimates for WTP in the preference space can be computed as follows:

$$WTP_{nk}=-\frac{\frac{dU}{dA_{nk}}}{\frac{dU}{d{cost}_{n}}}=\frac{\beta_{nk}}{\beta_{n,cost}}$$

where $\beta_{nk}$ is the coefficient of the attribute, while $\beta_{n,cost}$ is the coefficient of the cost attribute (i.e., test cost). The previous formula produces valid WTP estimates assuming the price variable as fixed. This is an obvious limitation because the price could have a random distribution in many cases.

To assess the robustness of the findings, a sensitivity analysis was conducted by handling straight-lining respondents.

#### 2.6.3. Re-Parametrised Model

Generally, the log-normal distribution describes a random price variable, although this would lead to unrealistic WTP values and heavily skewed distributions [42]. Therefore, we estimated the mixed logit model in the WTP space after performing the following re-parametrisation:

$$U_{ntk}=-\beta_{ncost}\left[c{ost}_{ntk}-\frac{1}{\beta_{ncost}}\sum_{k=1}^{4}\beta_{nk}A_{ntk}\right]+\varepsilon_{ntk}=-\beta_{ncost}\left[{cost}_{ntk}-\sum_{k=1}^{4}{WTP}_{nk}A_{ntk}\right]+\varepsilon_{ntk}$$

In this framework, the coefficients of the attributes are re-parametrised by taking the ratio between each attribute parameter and the attribute price (see Train and Weeks [37,44]). This transformation allows us to assume a distribution directly for ${WTP}_{nk}$ and not for the original coefficients. We assume each ${WTP}_{nk}$ to be normally distributed and $\beta_{ncost}$ to be log-normally distributed. Preference heterogeneity related to observable characteristics such as experience and occupation was assessed only in the preference space, while the assessment in the WTP space was not possible due to convergence issues. Following the same methodology, WTT was estimated both in preference and in WTT space, allowing interestingly for elicit trade-off preferences using a non-monetary variable (i.e., diagnostic yield) as a benchmark attribute.

Additionally, the mixed logit model was used to estimate the distributions of individual-level coefficients for each attribute following the approach proposed by Revelt and Train [45]:

$$\hat{\beta_{n}}=\frac{\frac{1}{R}\sum_{r=1}^{R}\beta_{n}^{\left[r\right]}\prod_{t=1}^{T}\prod_{j=1}^{J}{\left[\frac{\exp\left(x_{njt}^{\prime }\beta_{n}^{\left[r\right]}\right)}{\sum_{j=1}^{J}\left(x_{njt}^{\prime }\beta_{n}^{\left[r\right]}\right)}\right]}^{y_{njt}}}{\frac{1}{R}\sum_{r=1}^{R}\prod_{t=1}^{T}\prod_{j=1}^{J}{\left[\frac{\exp\left(x_{njt}^{\prime }\beta_{n}^{\left[r\right]}\right)}{\sum_{j=1}^{J}\left(x_{njt}^{\prime }\beta_{n}^{\left[r\right]}\right)}\right]}^{y_{njt}}}$$

where $\beta_{n}^{\left[r\right]}$ is the r-th draw for individual *n* from the estimated distribution of *β*.

In conclusion, part-worth utility values were computed by estimating a random effects multinomial logit model [46], which allows for obtaining the probability $P_{t}$ of a health professional choosing an alternative *t*, among a set of possible alternative *T*s in the choice task, with the *βs* representing the estimated part-worth utilities.

$$P_{t}=\frac{exp\left(\beta ,X_{t}\right)}{\sum_{j=1}^{T}exp\left(\beta ,X_{j}\right)}$$

A positive part-worth utility suggests that the given attribute level is preferred over levels of the same attribute. In contrast, larger part-worth utilities, with respect to smaller ones, indicated a higher degree of preference for one level over another. The computed part-worth utilities were then standardised to have a mean value of zero and used to calculate the attribute relative attribute importance (RI). Accordingly, RI was computed as:

RI=(overall utility for each attribute)/(total utility)

The overall utility for each attribute was given by the range of part-worth utilities within each attribute, whilst the total utility equalled the sum of the overall utility values among all the DCE attributes. The RI was expressed as a percentage. All statistical analyses were performed in STATA 17 (StataCorp LP, College Station, TX, USA).

## 3. Results

### 3.1. Respondents’ Characteristics

Overall, 200 surveys were sent out. A total of 125 eligible participants provided informed consent and finished the DCE survey. Thirty participants did not respond to multiple questions, and 14 straight-lined their responses. Hence, these participants were excluded from the analyses, resulting in a final sample with 81 respondents. Demographic characteristics are reported in Table 2. Women represented 55.8% of the sample, while most respondents were between 25 and 34 years old (39.7%).

### 3.2. Model of Choice Behaviour

Each respondent answered 10 choice tasks, so 810 choice tasks were completed. Table 3 illustrates the results of the mixed logit model.

The estimated coefficients show that NGS tests are preferred when they provide a higher diagnostic yield, require shorter counselling periods, lower costs, lower turnaround time, and identify fewer variants of unknown significance. All the estimated coefficients were of the expected sign and were statistically significant. Among the parameters, diagnostic yield generated the highest utility (coef. = 0.205). The coefficients of counselling time, test cost, turnaround time, and ability to identify variances of unknown significance were negative, implying a decrease in utility as the number of minutes of counselling, the cost, the number of weeks, and the probability of identifying variances of unknown significance increase, respectively.

Since the coefficients associated with the standard deviations are significant for attributes such as counselling time, diagnostic yield, turnaround time, and the ability to identify variants of unknown significance, this means that there is considerable heterogeneity around these parameters, and for this reason, we considered them as random.

With regard to heterogeneous effects, respondents with higher experience (i.e., >20 years) assigned lower levels of utility to the diagnostic yield attribute, i.e., 38% lower (i.e., –0.090/0.234) (Supplementary Text S3, Table S1, panel A) as per the average respondent. Regarding the job occupation, the coefficient associated with being a paediatrician is not statistically significant, implying no difference between being a medical geneticist and a paediatrician in terms of perceived utility of diagnostic yield. Conversely, biologists assigned higher levels of utility to the diagnostic yield attribute. Concerning medical geneticists, the utility assigned to the attribute is 100% more (i.e., 0.204/0.204) (Supplementary Text S3, Table S1, panel B).

Supplementary Text S4, in the Supplementary Materials, shows the distribution of individual-level coefficients for each attribute and provides a visual description of the parameters’ dispersion due to heterogeneous preferences. Of note are a few observations: (a) all the attributes manifested a heterogenous distribution; (b) diagnostic yield coefficients showed a right-skewed bimodal distribution; (c) the density curve of the turnaround time coefficients was concentrated around the mean having no skew; and (d) the remaining attributes’ coefficient density curves depicted unimodal right-skewed distributions.

Sensitivity analysis showed that coefficients and their significance level were largely consistent with the original estimates, with minor variations in the magnitude of preferences.

### 3.3. Willingness to Pay and Willingness to Trade Estimates

Table 4 reports the WTP estimates in preference and the WTP space. All attributes were assumed to be independent. Diagnostic yield, counselling time, and test cost were assumed to be fixed, while turnaround time and the ability to identify variances of unknown significance were random and log-normally distributed.

The WTP results in the preference space differed notably from those computed in the WTP space, with the latter showing larger values. Overall, the relevance of the proposed analysis to obtain unbiased estimates of the WTP appears justified.

In the preference space, the WTP estimates varied from EUR 387 (95% CI, 271.8–502.9) for a 7% increase in the diagnostic yield to EUR 469 (95% CI, 287.2–744.9) for a decrease of one week in the turnaround time. Furthermore, individuals with higher professional experience showed a WTP of 43% (i.e., −EUR 203/EUR 471) lower than those with less experience. Regarding the job occupation, biologists highlighted a WTP 76% (i.e., EUR 357/EUR 471) higher, with respect to medical geneticists, for a 7% increase in diagnostic yield (Supplementary Text S3, Table S2). In the WTP space, the WTP estimates varied from EUR 710 (95% CI, 427.5–992.1) for a 7% increase in the diagnostic yield to EUR 777 (95% CI, 752.4–802.2) and for a one-week decrease in the turnaround time.

Overall, the WTP for respondents was more significant for the investigation process and the waiting time, whereas the WTP for health professionals spending enough time in consultation was smaller.

Table 5 outlines the WTT estimates in preference and the WTT space.

Contrary to the WTP, the WTT results in the preference space were similar to those in the WTT space. In both the preference and the WTT spaces, turnaround time is associated with the highest coefficient. Responders were willing to reduce diagnostic yield by 1.2 or 1.1 units (corresponding to a 7% reduction) to decrease the turnaround time by one week in both the preference and the WTT spaces, respectively. They were willing to reduce diagnostic yield by 0.07 and 0.06 percentage points to increase counselling time by one hour. Finally, they were willing to reduce the diagnostic yield by 0.08 and 0.05 percentage points to increase the ability to detect variances of unknown significance by one percentage point.

### 3.4. Relative Importance of Attributes

**Figure 1:**
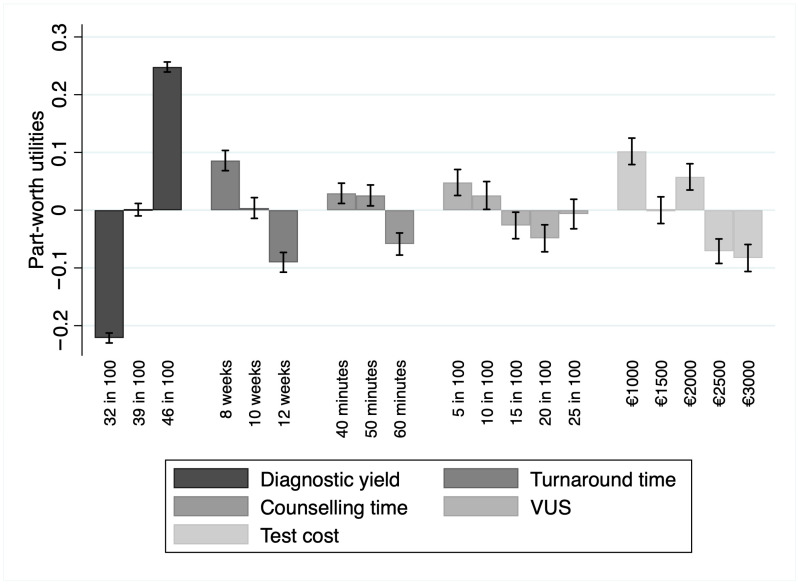
Part-worth utility values for each attribute level alongside their 95% confidence intervals. The bars represent the estimated utilities, while the error bars indicate the uncertainty around these estimates. Abbreviation: VUS, variants of unknown significance. Artwork created with STATA 17 (StataCorp LP, College Station, TX, USA).

The relative importance demonstrated that, among the attributes, respondents valued diagnostic yield as the most important (RI = 50.36%; 95% CI 40.2–67.2%), preferring tests with higher values of this attribute. In the context of this study, the second-most important attribute was the turnaround time (RI = 18.92%; 95% CI 14.4–25.9%). Participants valued a lower turnaround time over a larger one. Other attributes, in order of relative importance, were test cost (RI = 10.93%, 95% CI 7–16.3%), the probability of detecting variances of unknown significance (RI = 10.37%; 95% CI 6.5–15.6%), and the counselling time (RI = 9.42%; 95% CI 6.2–13.9%). Part-worth utilities were in the expected direction for the levels within each of the above-reported attributes, with more extensive preferences for higher counselling time, lower probability of detecting variances of unknown significance, and lower costs. Part-worth utilities are depicted in Figure 1.

## 4. Discussion

We evaluated the attributes’ burden to detect specialists’ preferences in the process of deciding on a testing strategy. We submitted to responders a hypothetical scenario in which a newborn infant presented a clinical picture compatible with a genetic disease. Referring to the previous case, we asked health specialists to provide their preferences regarding WTP, diagnostic accuracy, waiting time, counselling time, and VUS identification. The resulting responses suggest that uptake for NGS tests was positively affected by a higher diagnostic yield, shorter periods of counselling, lower costs, shorter turnaround time, and identifying fewer variants of unknown significance.

Our findings show that the most important attribute was the diagnostic yield, for which responders were willing to spend up to EUR 387 for a 7% increase in accuracy. This result is relevant since the test accuracy is fundamental for diagnosing genetic disorders. Based on its diagnostic yield, the genetic test choice could directly support the diagnosis process, affecting health professionals and their clinical management decisions [47]. Moreover, a genetic disease, disorder, or phenotype can be challenging to define, considering the possible technical error derived from an outdated instrument [48]. Indeed, an uncertain result might require an additional exam, resulting in higher costs and time loss [49].

A further remarkable finding was the importance of a shorter turnaround time. This likely reflects that rapid identification of genetic diseases may provide information to direct clinical and public health interventions to shorten or end the diagnostic odyssey [50]. As is known, the impact of rapid genomic testing on morbidity and mortality through immediate access to provisional diagnosis, for which a specific treatment might eventually be available, has already been widely described in the literature [50].

The DCE methodology is not a novelty in the clinical field as well. Indeed, its application involved different scenarios of the care process [43,51,52]. This study proposes a novel application of the DCE methodology to the fast-growing genomic diagnostic technology field, considering that further investigation is needed in this context. Our estimates match previous findings in other DCE studies evaluating the preferences of all the actors involved in the diagnosis process (e.g., patients, health professionals, and payers). In the genetic testing context, Buchanan et al. supported the findings that health professionals mostly valued the diagnostic yield and the ability to identify fewer variances of unknown significance compared to counselling time, which instead has been less prioritised [21]. In addition, a systematic review of the literature strengthened this result [53].

Our study aligns with a common finding establishing the greater preference of health professionals for outcome attributes (i.e., diagnostic yield and identification of variances of unknown significance) with respect to process outcomes (i.e., counselling time). Ries et al. [54] reported similar findings regarding WTP for genetic testing, albeit from a different point of view. Here, respondents from the general population were willing to invest more than CAD 500 for genetic diagnostic services. Additionally, a literature review summarising median WTP values for different diagnostic technologies identified a median WTP ranging from USD 100 to USD 1000 for genetic testing [55]. With reference to the assumed importance of adopting NGS tests for an early diagnosis and treatment, evidence in the scientific literature reinforces this hypothesis even from a payer perspective. Dhanda et al. stated that payer-preferred genetic tests improving the quality of life had high expert agreement on changing medical care and increased life expectancy through targeted treatments [22].

The study finding that health professionals with over 20 years of experience placed less emphasis on diagnostic yield aligns with patterns observed in the broader literature [56,57]. This perspective is supported by studies examining the interplay between clinical experience and decision making. Nalliah et al. has shown that intuition-based decision making, under certain conditions, can be as effective or even superior to evidence-based processes. These conditions include the presence of domain expertise, typically requiring several years of practice beyond initial qualification, as well as scenarios with time constraints, complex and ambiguous problems, or limited scientific evidence. In such cases, intuition allows experts to rapidly access subconsciously stored knowledge, enabling quick and effective decision making [58]. In addition, the balance between experience-based and evidence-based approaches is increasingly recognised in clinical practice. Wattiez et al. emphasised that experience-based management can complement evidence-based guidelines by integrating tacit knowledge and insights gained from real-world practice into decision-making processes. This integration is particularly valuable in addressing complex or multifaceted conditions, where guidelines may not account for every clinical scenario [59]. In the context of genomic diagnostics, experienced clinicians may develop a more nuanced perspective on the diagnostic process, recognising that diagnostic yield, while an important metric, does not always guarantee better patient outcomes. These individuals might prioritise other factors such as clinical judgement and patient context as well as on long-term clinical outcomes, resource utilisation, or broader implications of diagnostic decisions, viewing diagnostic yield as just one component of overall care quality [60].

Considering the elicited preferences from health professionals, our findings may steer decision making toward the choice of NGS tests, which should ensure a higher diagnostic yield and a shorter turnaround time. The joint reading of our findings allows for some practical main implications. The strong preference for shorter turnaround times underscores the importance of policies promoting process optimisation in genomic diagnostic workflows. Healthcare policies should prioritise investments in infrastructure and technology that expedite the analysis and interpretation of genomic data, potentially integrating automated pipelines and artificial intelligence tools. For instance, Australia’s Medicare Benefits Scheme introduced reimbursement policies for genomic testing in rare diseases and cancer, enabling faster access to testing and improving patient outcomes. Similarly, the UK’s National Health Service Genomic Medicine Service implemented a centralised approach to genomic diagnostics, providing equity of access to high-quality NGS services while streamlining turnaround times. Furthermore, the significant role of test cost in shaping clinician preferences highlights the necessity of establishing economically sustainable policies focused on implementing reimbursement systems for genomic diagnostics technologies. For example, Germany introduced statutory health insurance coverage for specific NGS panels, reducing financial barriers and fostering adoption among clinicians. Moreover, the differences in preferences between experienced and less experienced professionals suggest that training programs should be tailored to address specific knowledge gaps. The emphasis on diagnostic yield across all respondents highlights the potential for genomic diagnostics to reduce inequities in healthcare. Policymakers should aim to integrate these technologies within underserved and resource-limited settings, ensuring that vulnerable populations can benefit from precision medicine advancements. A more conceptual implication may be derived from comparing our findings with those obtained in different contexts as reported by the scientific literature. Indeed, it is paramount to give accountability to all the actors engaged in the diagnostic workup when developing sounding genomic policies, especially by weighing health professionals’ preferences with patients’ and payers’. This may address two issues about empowerment. Firstly, it may guarantee to overcome the shattering relationship [61,62] among the stakeholders involved at the different levels (i.e., macro-, meso-, and micro-level) of the decision-making process in the healthcare system. Secondly, it might optimise the health system performance by pursuing the Triple Aim framework [63], which embodies the core principles of public health, by simultaneously improving population health experience of care, and decreasing the burden of healthcare costs for national health services.

The present study should be considered considering its main strengths and limitations. Given the online nature of the experiment, participation and non-response biases are potential caveats to data collection [64]. Moreover, the personal experience may influence the attitude of the responder towards the items. Nevertheless, the web survey was addressed only to sector specialists, thus increasing the likelihood of obtaining coherent answers. Additionally, the time needed to complete the questionnaire might have discouraged some recipients, potentially reducing the sample size. In addition, the observed considerable heterogeneity was not explored in depth due to the study’s sample size. However, proper methodology [33,34] was adopted to address this issue by computing the needed sample to yield reliable estimates of the attributes’ coefficients. It has been noted that a sample size of at least 20 is sufficient to correctly compute a DCE model since each participant provides multiple observations [65]. A further limit was that a reduction in the amount of counselling time positively affected the uptake of genetic tests. Nonetheless, this may be an artefact of the design of the DCE, even though the relative size of the coefficients for the counselling attribute in the regression model may imply that this attribute likely only had a negligible impact on the individuals’ choices. A further caveat is the presence of missing data that can significantly affect the study findings by introducing bias, reducing statistical power, and impairing the validity of preference estimates. Notwithstanding, a sensitivity analysis confirmed that the exclusion of missing data did not materially affect the parameter estimates, ensuring the robustness of the results. An additional limitation is the exclusion of an opt-out option, which might have introduced some bias on preference estimation, realism, behavioural responses, and generalisability.

Excluding an opt-out option forces respondents to select one of the available alternatives. This can increase the precision of utility estimates for the attributes under consideration, as all responses contribute information about preferences. It may also lead to biased estimates if respondents would have preferred not to choose either option in a real-world scenario, thus artificially inflating the importance of certain attributes.

As the study is based on a real-world setting where clinicians must choose between two genomic tests, excluding the opt-out option increases the contextual realism of the study. Notwithstanding, without an opt-out option, respondents might make choices they would not make in practice, leading to hypothetical bias.

Forcing respondents to choose between two alternatives in every choice set can lead to decision fatigue, resulting in lower engagement, random answering, or reduced reliability of responses. Therefore, the increased variability in responses might reduce the precision of the estimated preferences.

The absence of an opt-out option may limit the generalisability of the findings to settings with different clinical workflows.

Notwithstanding, the study did not adopt an experimental design with alternative-specific attribute levels nor a labelled design, allowing it to achieve the level balance for all attributes and to improve response efficiency [66]. Furthermore, including an opt-out option could misrepresent the actual clinical workflow, potentially leading to responses that deviate from such real-world practice. Finally, in the present DCE, responders answered several choice tasks while assessing a hypothetical scenario. Alternative scenarios might originate different patterns of results.

The observed differences between preference space and WTP space estimates can be attributed to several factors inherent to the modelling approaches. First, preference space models estimate utility coefficients directly for each attribute, while WTP space models reformulate utility functions to estimate WTP by dividing attribute coefficients by the marginal utility of cost. This transformation introduces additional assumptions about the distribution of the cost parameter, which can differ between the two approaches. Variability or non-linear scaling in the cost parameter, such as declining marginal utility with increasing cost, may further amplify disparities. Additionally, WTP space estimates are more sensitive to unobserved heterogeneity in cost sensitivity, as variability in the cost parameter directly affects WTP estimates for all non-monetary attributes.

Further research is required to investigate the drivers of preference heterogeneity among health professionals about genomic testing through revealed preference studies.

Additional research should aim to include a more diverse range of professional backgrounds, such as genetic counsellors and other specialised healthcare providers, to enable more granular comparisons of preferences across subgroups.

## 5. Conclusions

The highly shared preference for the diagnostic yield and turnaround time draws attention to the need to invest in more accurate and faster instruments to tackle the diagnostic odyssey of ill paediatric patients. Study findings have implications for the design of future genomic policies and the implementation of genomic testing services. These preferences may also impact the translation of NGS techniques into clinical practice for diagnosing suspected genetic disorders in the medium-to-long term. Investigating NGS application to diagnose suspected genetic disorders through sector specialists’ perspectives may generate room for accurate and early genetic diagnosis, ultimately reducing the diagnostic odyssey [67,68]. However, sustainable allocation of healthcare resources should become a focal point in discussing economic investments in the genetic field to ensure the most suitable and cost-effective care for the fragile paediatric population.

## Figures and Tables

**Table 1:** Summary of attributes and levels adopted in the final DCE survey.

Test Attribute	Possible Levels for Each Testing Alternative	
	Genetic Test A	Genetic Test B
Diagnostic yield	Pathogenic variation identified in 32 out of 100 cases	
	Pathogenic variation identified in 39 out of 100 cases	
	Pathogenic variation identified in 46 out of 100 cases	
Turnaround time	8 weeks, 10 weeks, 12 weeks	
Counselling time	40 min, 50 min, 60 min	
Ability of the test to identify variants of unknown significance	Variants of unknown significance identified in 5 out of 100 cases	
	Variants of unknown significance identified in 10 out of 100 cases	
	Variants of unknown significance identified in 15 out of 100 cases	
	Variants of unknown significance identified in 20 out of 100 cases	
	Variants of unknown significance identified in 25 out of 100 cases	
Test cost	EUR 1000, EUR 1500, EUR 2000, EUR 2500, EUR 3000	

**Table 2:** Demographic characteristics of DCE survey participants.

Variable	Value	SD
Gender		
Female	55.8%	0.51
Male	41.7%	0.49
Prefer not to say	2.5%	0.16
Age of respondent		
24 years and under	2.5%	0.16
25–34 years	39.7%	0.49
35–44 years	13.7%	0.34
45–54 years	8.7%	0.28
55–64	19.9%	0.39
65 years and over	15.5%	0.36
Respondent occupation		
Clinical geneticist	34.8%	0.48
Paediatrician	45.3%	0.49
Biologist	17.4%	0.38
Laboratory scientist	1.25%	0.11
Other	1.25%	0.11
Respondent’s experience in the genomic field		
Less than 1 year	37.4%	0.48
1–9 years	33.5%	0.47
10–20 years	18.1%	0.39
20 years and over	11%	0.31
Education/research activities on NGS testing approaches		
Yes	44.6%	0.49
No	55.4%	0.49
Use of NGS testing approaches		
Yes	44.8%	0.49
No	55.2%	0.49
Survey details		
Median time to complete the survey, minutes	12.1	3.16
Survey response rate	62.2%	0.03

Abbreviation: NGS, next-generation sequencing.

**Table 3:** Mixed logit regression estimates.

Attribute	B-Coefficient	SD	Lower CI	Upper CI	*p*
Fixed parameters					
Diagnostic yield	0.205 ***	0.659	0.178	0.232	<0.001
Counselling time	−0.015 *	0.326	−0.028	−0.001	<0.071
Test cost	−0.001 ***	0.039	−0.0007	−0.0004	<0.001
Random parameters					
Turnaround time	−0.248 ***	2.050	−0.339	−0.173	<0.001
Variance of unknown significance	−0.016 ***	0.511	−0.037	0.004	<0.001
AIC	782.29				
Log-likelihood	−384.15				

Abbreviations: AIC, Akaike Information Criterion; CI, 90% confidence interval; *p*, *p*-value; and SD, standard deviation. Notes: significant levels: *** *p* < 0.01, and * *p* < 0.1.

**Table 4:** Comparison of willingness to pay estimates in preference and WTP space.

Attribute	Preference Space	WTP Space
	Estimate (CI 95%)	Estimate (CI 95%)
Diagnostic yield	EUR 387.3 (271.8–502.9)	EUR 709.8 (427.5–992.1)
Turnaround time	EUR 468.7 (287.2–744.9)	EUR 777.3 (752.4–802.2)
Counselling time	EUR 27.8 (2.5–58.8)	EUR 44.9 (12.3–77.6)
Variance of unknown significance	EUR 31.2 (8.6–77.3)	EUR 42.4 (39.01–45.8)

Abbreviation: CI, confidence interval. Notes: Those attributes for which increasing values correspond to undesired situations by the health professional (i.e., turnaround time, counselling time, and ability to detect variance of unknown significance) are associated with negative coefficients in the mixed logit model providing negative WTP values. However, to ease the interpretability of the results, WTP estimates related to the abovementioned attributes were multiplied by −1 and converted into positive values. Narrow CIs indicate greater precision in the estimate, suggesting a high degree of agreement among respondents regarding the value of the attribute. Wider CIs reflect higher uncertainty, which may result from variability in respondents’ preferences. Preference space estimates assume a fixed cost coefficient, providing stable and narrower confidence intervals for WTP by dividing attribute coefficients by the cost coefficient. In contrast, WTP space estimates model the cost coefficient as random, accounting for variation in cost sensitivity across respondents. While WTP space estimates reflect heterogeneity more realistically, they often exhibit wider confidence intervals and greater variability. Differences between the two approaches highlight the extent of heterogeneity in cost preferences among respondents and should be interpreted accordingly.

**Table 5:** Comparison of willingness to trade estimates in preference and WTT space.

Attribute	Preference Space	WTT Space
	Estimate (CI 90%)	Estimate (CI 90%)
Turnaround time	1.21 (0.786–1.677)	1.09 (1.022–1.158)
Counselling time	0.07 (0.006–0.138)	0.06 (0.009–0.117)
Variance of unknown significance	0.08 (−0.021–0.186)	0.05 (0.022–0.094)

Abbreviation: CI, confidence interval. Notes: Those attributes for which increasing values correspond to undesired situations by the health professional (i.e., turnaround time, counselling time, and ability to detect variance of unknown significance) are associated with negative coefficients in the mixed logit model providing negative WTP values. However, to ease the interpretability of the results, WTP estimates related to the abovementioned attributes were multiplied by −1 and converted into positive values.

## Data Availability

The datasets generated during and/or analysed during the current study are available from the corresponding author upon reasonable request.

## Supplementary Materials

The following supporting information can be downloaded at: https://www.mdpi.com/article/10.3390/jpm15010025/s1, Supplementary Text S1: Literature review; Supplementary Text S2: DCE attributes and hypothetical choice situation presented; Supplementary Text S3: Model of choice behaviour and WTP estimates considering heterogeneous effects; Table S1: Mixed logit regression estimates for heterogenous effects; Table S2: WTP estimates in preference space for heterogenous effects; Supplementary Text S4: Distributions of individual-level coefficients; Figure S1: Density function—individual-level coefficients for the “Diagnostic yield” attribute; Figure S2: Density function—individual-level coefficients for the “Turnaround time” attribute; Figure S3: Density function—individual-level coefficients for the “Counselling time” attribute; Figure S4: Density function—individual-level coefficients for the “Ability of the test to identify variants of unknown significance” attribute; Figure S5: Density function–individual-level coefficients for the “Test cost” attribute.
