# Supplementary material for: Health Professionals’ Preferences for Next-Generation Sequencing in the Diagnosis of Suspected Genetic Disorders in the Paediatric Population
Source: J Pers Med. 2025 Jan 10;15(1):25. doi: 10.3390/jpm15010025 (PMC11766785; doi:10.3390/jpm15010025)

Health Professionals' Preferences for Next-Generation Sequencing in the Diagnosis of Suspected Genetic Disorders in the Paediatric Population

Supplementary materials

Contents

Supplementary Text S1: Literature review
Supplementary Text S2: DCE attributes and hypothetical choice situation presented
Supplementary Text S3: Model of choice behaviour and WTP estimates considering heterogeneous effects
Supplementary Text S4: Distributions of individual-level coefficients

Supplementary Text S1: Literature review

Methods

A literature review was performed in order to collect and investigate papers providing information on the factors leading clinicians to choose NGS as a diagnostic instrument for newborns. A search string was applied to the main online literature databases, namely Pubmed, Embase, Scopus and Genereviews, including the following terms:

("discrete choice *" OR “DCE” OR “discrete choice experiment”) AND ("screening" OR “ICU screening” OR “Intensive Care Unit screening” OR “neonatal screening” OR “neonatal test” OR “newborn screening” OR “newborn test” OR “infant screening” OR “infant test”) AND (“Next Generation Sequencing” OR “NGS” OR “Whole genome sequencing” OR “WGS” OR “Whole Exome Sequencing” OR “WES”).

Chosen inclusion criteria were the following:

- Articles published on a peer-reviewed journal
- Written in English language
- Reporting clinicians experience
- Focusing on NGS technologies

The literature search was undertaken between January 2022 and June 2022. After clearing articles from duplicates, the remaining were screened for title and abstract and articles quality was assessed. The selected articles' full texts were then evaluated regarding coherence with the search query and relevance. Finally, data were extracted from included publications.

Results

The database search identified 462 papers. Four articles were initially removed as duplicates. The screening for title and abstract reduced the number of potentially includible articles to a total of 92. The full text analysis brought to a total of 11 included articles.

A total of 5 attributes were considered of significant importance in terms of our assessment. Of these attributes, the economic impact of the evaluated tests was the most recurrent among the selected articles (9 out of 11), followed by the number of identified pathogenic mutations (7 out of 11) and elaboration time (5 out of 11). All 5 attributes were included in the final discrete choice experiment addressed to health professionals.

Supplementary Text S2: DCE attributes and hypothetical choice situation presented

Test attributes – Attribute 1
Diagnostic yield
The likelihood that a test or procedure will provide the information needed to establish a diagnosis.

Test attributes – Attribute 2
Counselling time
The time the specialist needs to provide advice and instructions to patients once receiving an examination report.

Test attributes – Attribute 3
Test cost
The amount of money directly related to the execution of an instrumental test incurred by the national health service.

Test attributes – Attribute 4
Turnaround time
The total time needed for an instrumental test, from composition of an order by the prescriber, through verification and processing, to report production.

Test attributes – Attribute 5
Variance of unknown significance
A genetic change whose impact on the individual's health risk is not yet known.

Hypothetical choice set presented to participants

A newborn with muscular hypotonia, after a negative muscular spinal atrophy test, was sent to the neonatal intensive care unit. Its parents stated that it was not able to latch on the mother's breast and it seemed to have an abnormal respiration. The parents had several previous miscarriages, and this is their first child. The pregnancy ended at term without any complications. Routine clinical check-ups of tone confirmed a poor control of the head and hypotonic limbs. The newborn is currently being assisted by artificial respiration and nutrition. Right now, two next-generation sequencing tests are available that could provide useful genetic information to the clinical management of the patient and its family.

**Attributes**	**Genetic test A**	**Genetic test B**
Diagnostic yield	Pathogenic variation is identified in 46 out of 100 every cases	Pathogenic variation is identified in 39 out of 100 every cases
Turnaround time	10 weeks	8 weeks
Counselling time	50 minutes	60 minutes
Ability of the test to identify variants of unknown significance	Variant of unknown significance is identified in 10 out of every 100 cases	Variant of unknown significance is identified in 15 out of every 100 cases
Test cost	€1500	€1000

Which genetic test would you choose for this patient?

Genetic test A	Genetic test B

Supplementary Text S3: Model of choice behaviour and WTP estimates considering heterogeneous effects

Table S1. Mixed logit regression estimates for heterogenous effects.

**Attribute**	**B-coefficient**	**SD**	**Lower CI**	**Upper CI**	***P***
**Panel A**					
Diagnostic yield	0.234***	0.839	0.199	0.268	< 0.001
Experience X Diagnostic yield	–0.090***	0.659	–0.143	–0.037	0.005
**Panel B**					
Diagnostic yield	0.204***	1.049	0.161	0.248	< 0.001
Paediatrician X Diagnostic yield	–0.044	1.297	–0.097	0.009	0.173
Biologist X Diagnostic yield	0.204***	2.779	0.09	0.318	0.003

Abbreviation: CI, confidence interval; SD, standard deviation; P, p-value

*Notes: Significant levels: *** p<0.01, ** p<0.05, * p<0.1*

Table S2. WTP estimates in preference space for heterogenous effects.

**Attribute**	**Preference space**
	Estimate (CI 95%)
Diagnostic yield	€471.5 (311.7 – 631.3)
Experience X Diagnostic yield	€203.4 (92.7 – 356.4)
Paediatrician X Diagnostic yield	€129.4 (23.2 – 262.5)
Biologist X Diagnostic yield	€357.1 (114.8 – 599.4)

Abbreviation: CI, confidence interval

Notes: Those attributes for which increasing values correspond to undesired situations by the health professional (i.e., turnaround time, counselling time, ability to detect variance of unknown significance) are associated to negative coefficients in the mixed logit model providing negative WTP values. However, to ease the interpretability of the results, WTP estimates, associated to the abovementioned attributes, were multiplied by -1, and consequently converted into positive values.

Supplementary Text S4: Distributions of individual-level coefficients

Fig. S1 Density function – individual-level coefficients for the “Diagnostic yield” attribute

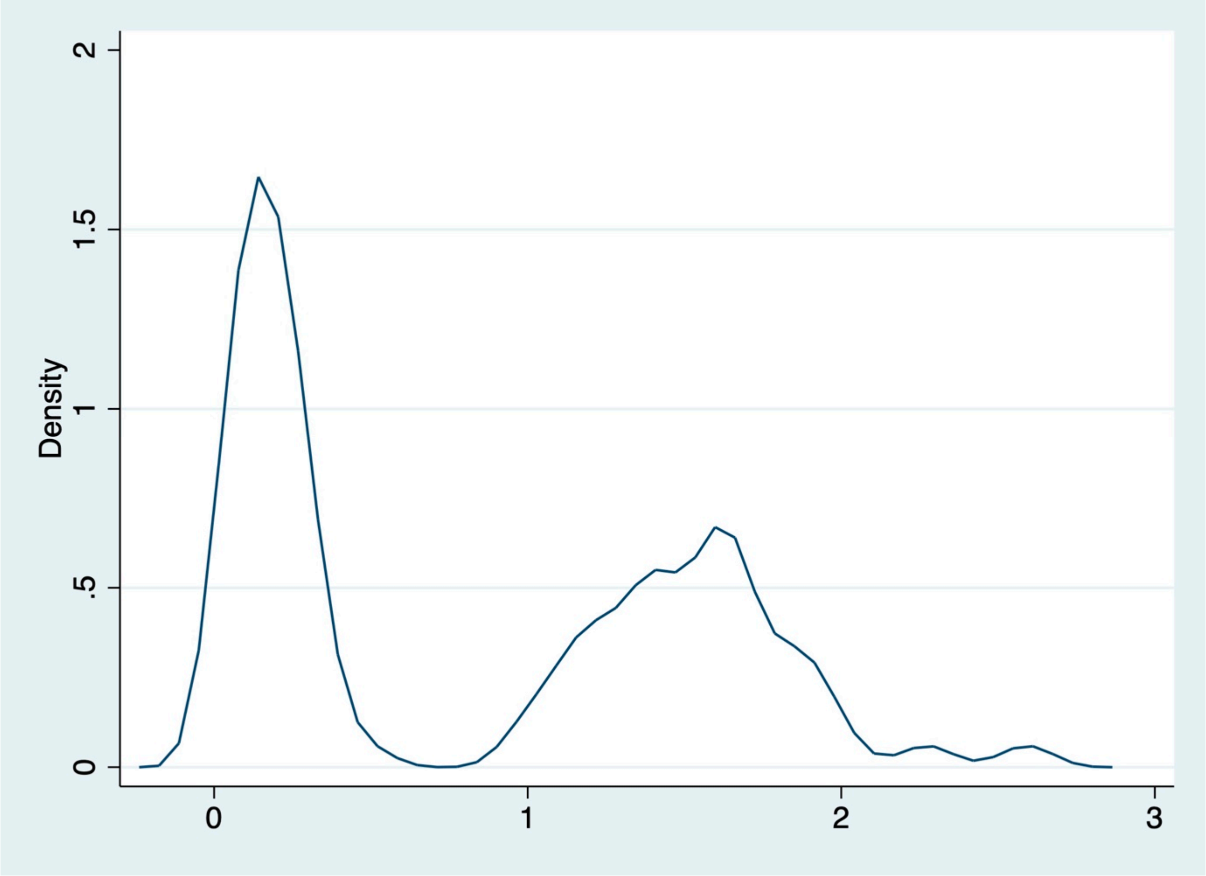

Fig. S2 Density function – individual-level coefficients for the “Turnaround time” attribute

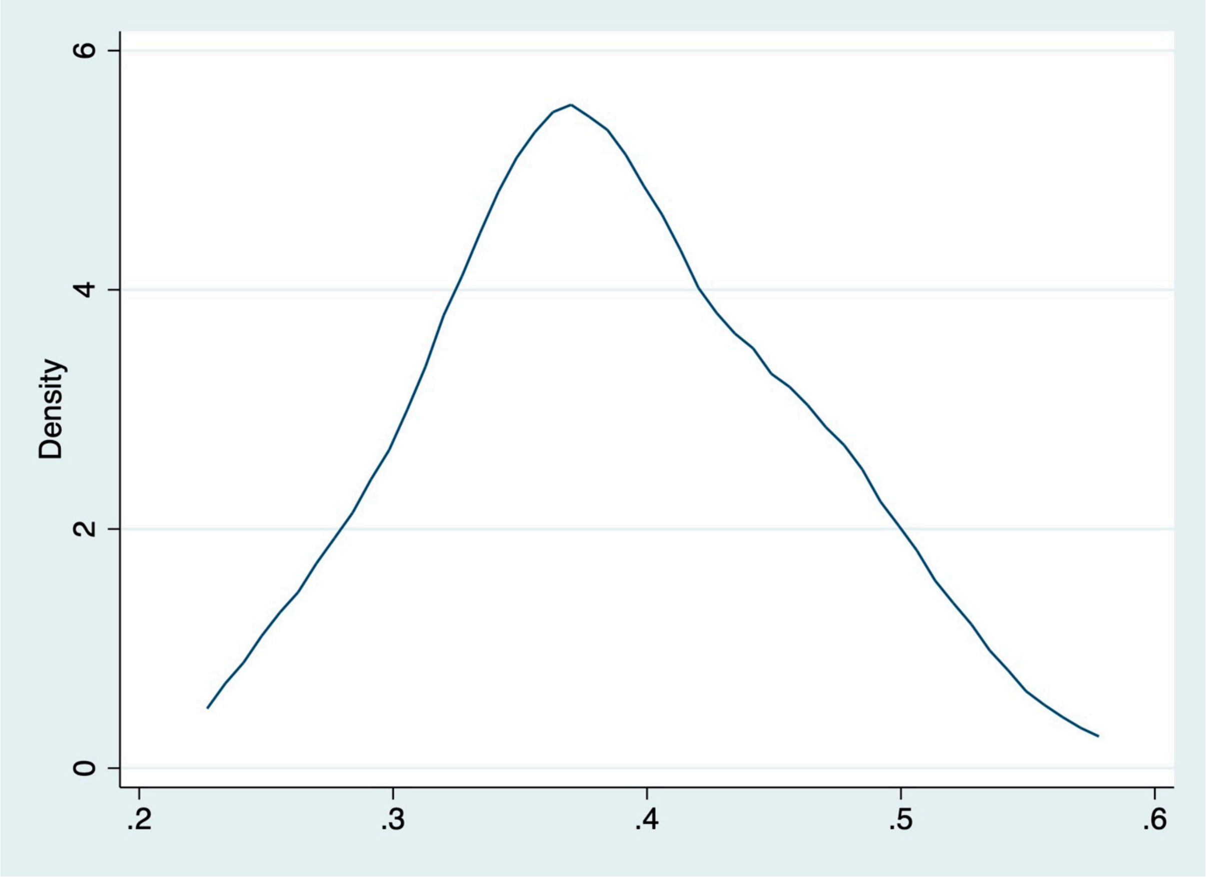

Fig. S3 Density function – individual-level coefficients for the “Counselling time” attribute

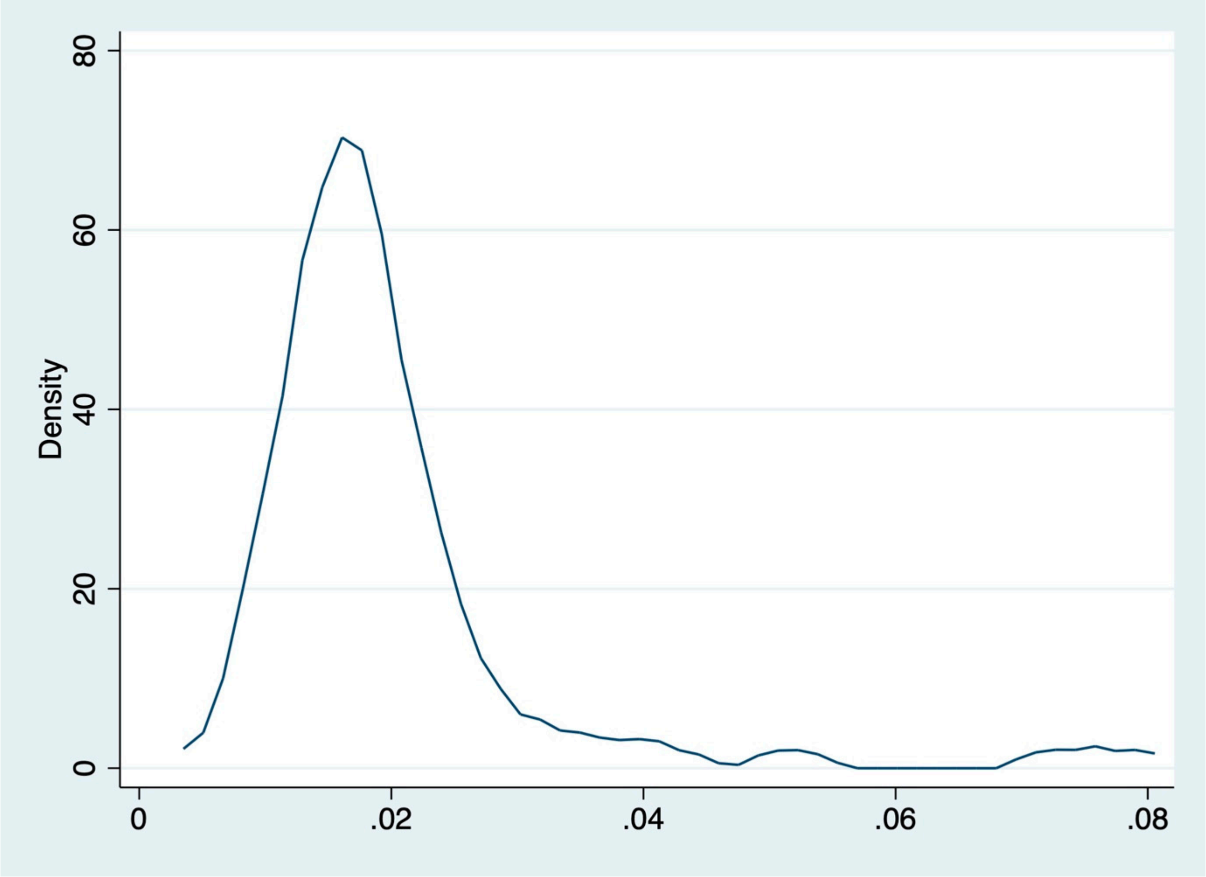

Fig. S4 Density function – individual-level coefficients for the "Ability of the test to identify variants of unknown significance" attribute

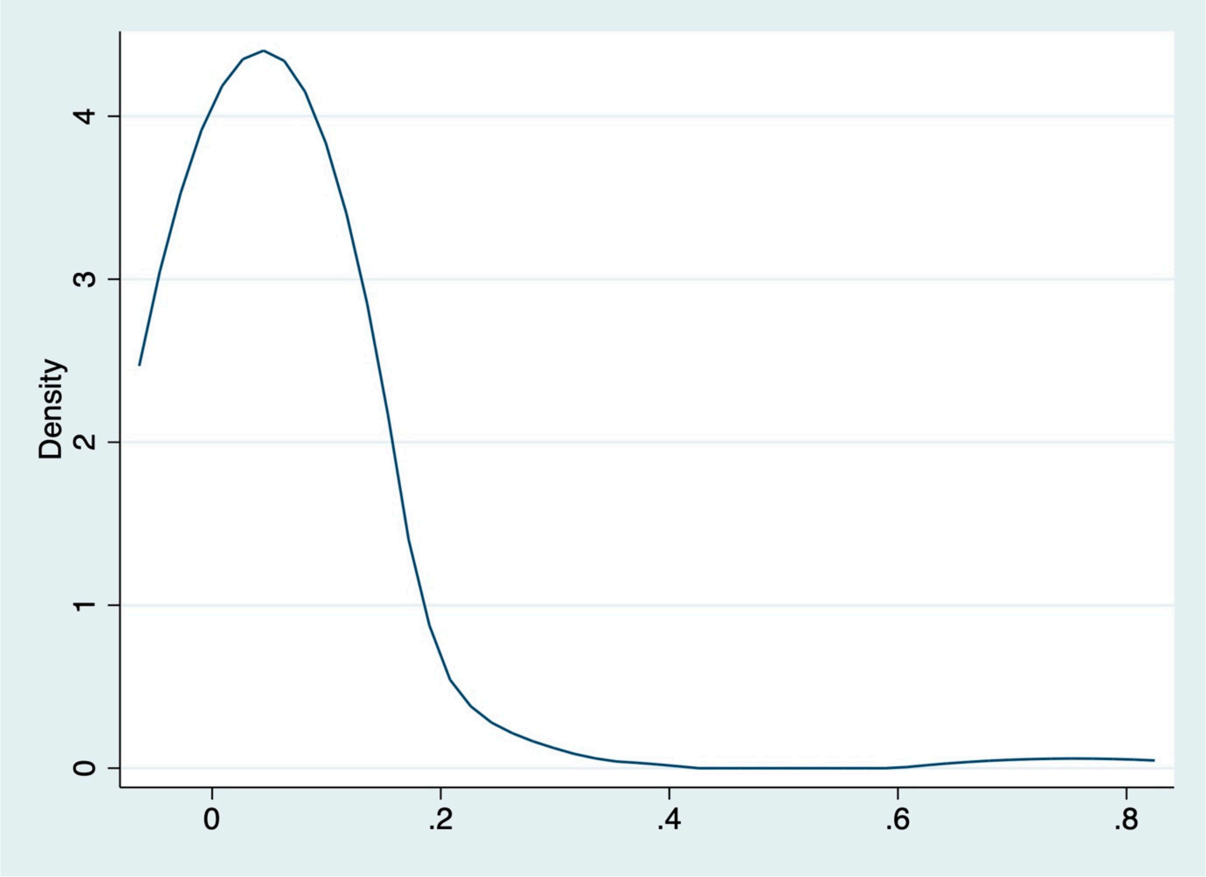

Fig. S5 Density function – individual-level coefficients for the “Test cost” attribute

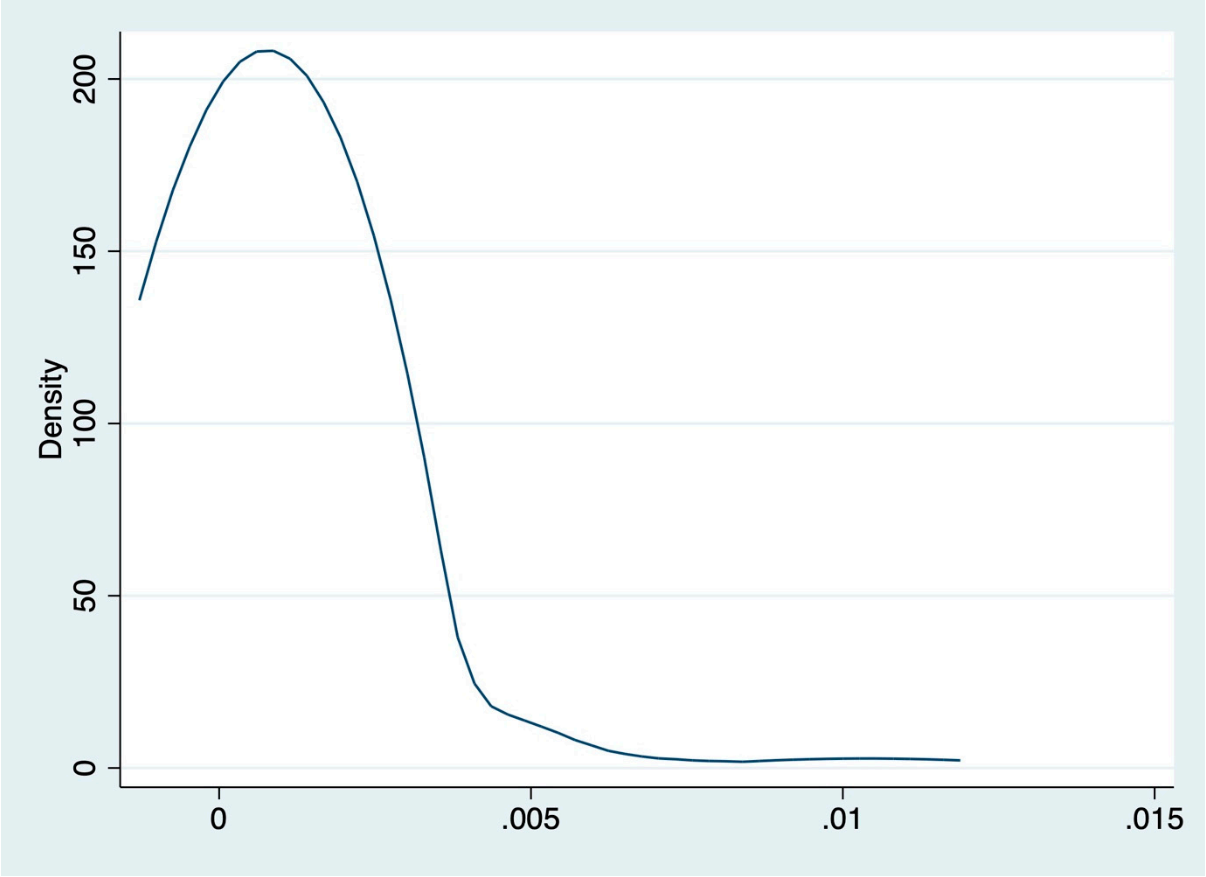

Supplement: Supplementary file 1 [file jpm-15-00025-s001.zip › jpm-3346478-supplementary.pdf]
